# Clinicopathological Features of Neuroendocrine Tumors in Gastroenteropancreatic Tract: A Single Center Study

**DOI:** 10.7759/cureus.27384

**Published:** 2022-07-28

**Authors:** Zubaria Rafique, Aafia Qasim, Asma Zafar, Seemal Ali, Akhtar S Chughtai, Aribah Atiq

**Affiliations:** 1 Histopathology, Chughtai Institute of Pathology, Lahore, PAK

**Keywords:** clinicopathological features, grading, neuroendocrine tumors, mixed neuroendocrine-non-neuroendocrine neoplasms, gastroenteropancreatic neuroendocrine tumors

## Abstract

Background: Gastroenteropancreatic neuroendocrine tumors (GEP-NETs) are a heterogeneous group of tumors with varying biological, functional, and clinical characteristics that develop from the gastroenteropancreatic tract's diffuse neuroendocrine system. The objective of this study is to determine the clinicopathological features of GEP-NETs at our facility.

Methodology: A cross-sectional analysis of 87 biopsies and resection specimens from January 2020 to January 2022 was performed. The histopathological reports as well as patient's demographic and clinic pathological data were obtained. Two pathologists with a special interest in gastroenteropancreatic pathology blindly reviewed all cases. The tumor grade and stage were determined using the WHO classification (2019) and the AJCC TNM system (8th edition). The data were analyzed with SPSS version 22 (IBM Corp., Armonk, NY, USA).

Results: Of the total 87 patients, 49 (56.3%) were male. The age range was 11 to 80 years, with a mean of 45.7±16.4 and the majority (56.3%) were under 50 years. The most frequent symptom was abdominal pain (55.2%). The most common site of GEP-NETs was the appendix (21.8%), followed by the ileum (18.4%), with the majority of tumors being non-functional (96.5%). Furthermore, neuroendocrine tumor (NET) grade 1 accounts for 62% of the total, followed by NET grade 2 (24.1%), neuroendocrine carcinoma (NET) grade 3 (10.3%), and mixed neuroendocrine-non-neuroendocrine neoplasms (MINENs) (3.5%). Synaptophysin was found to be positive in 83.9% cases while Chromogranin A was positive in 39.1%. A pathologic tumor (pT) stage was determined in 47 resection specimens in our study and the most common stage was pT3 (36.1%). Nodal metastasis was found in 25.5% of patients.

Conclusions: According to our study, appendix and ileum were the most common GEP-NETs sites. The tumor site and grade were shown to significantly correlate among the clinicopathological features but there was no discernible correlation between the tumor grade and the gender, age, or pathological tumor (pT) stage.

## Introduction

Gastroenteropancreatic neuroendocrine tumors (GEP-NETs) are a diverse group of neoplasms that arise from diffuse neuroendocrine cells and range from indolent well-differentiated neuroendocrine tumors to aggressive poorly differentiated neuroendocrine carcinomas. [1]. Neuroendocrine tumors can be found in various sites throughout the body with GEP-NETs making up more than half of them [2]. These tumors exhibit heterogeneous biological, functional, and clinical characteristics. They account for 2% of all malignant tumors; however, in the recent past, their incidence has increased due to endoscopic screening and early detection [3]. In Pakistan, there is a scarcity of data on the incidence of neuroendocrine tumors. The annual cancer registry by a local hospital in Punjab states 5.2% incidence while 2.5% incidence was reported from an institute based in Sindh Province [4].

Pathological parameters of tumor differentiation, grading, and staging determine the prognosis and management of GEP-NETs [5]. The clinical and pathologic characteristics of these tumors need to be studied in order to better comprehend the variation in location, grade, and behavior. Overall little research on the incidence and grade of these tumors has been conducted both at the national and international levels [6].

In the current study, clinicopathological characteristics of GEP-NETs diagnosed on biopsy or resection specimens were gathered to assess the relationship of tumor grade with the clinical and pathological parameters comprising age, gender, anatomical site, and pathologic tumor (pT) stage.

## Materials and methods

This was a cross-sectional descriptive study conducted between January 2020 and January 2022 with approval from the Institutional Review Board (IRB) Committee of the Chughtai Institute of Pathology, Lahore, Pakistan (Reference letter no. CIP/IRB/1056). Total 87 diagnosed cases of neuroendocrine neoplasms were retrieved from archives of Chughtai lab using Nexus software. In cases with both preoperative diagnostic biopsies and resection specimens, only resection biopsies were included in the study. Metastatic neuroendocrine tumor cases (liver deposits) and goblet cell adenocarcinoma of appendix were excluded. Patient’s demographic and clinicopathological data, including age, gender, tumor location, clinical features, Ki-67 proliferation index, and mitotic counts, were recorded.

**Figure 1:**
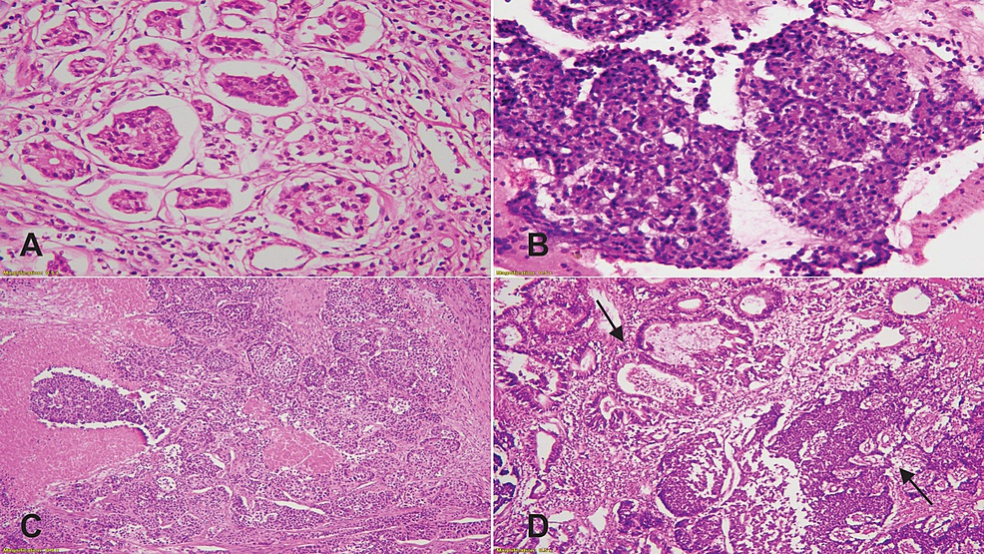
(A) H&E image at 400x magnification shows nested pattern in NET grade 1. (B) H&E image at 100x magnification shows NET grade 2. (C) H&E image at 40x magnification shows poorly differentiated tumor with necrosis in NEC grade 3. (D) H&E image at 40x magnification shows adenocarcinoma and neuroendocrine components in MINEN (black arrows). H&E: hematoxylin and eosin; NET: neuroendocrine tumor; NEC: neuroendocrine carcinoma; MINEN: mixed neuroendocrine-non-neuroendocrine neoplasm.

**Figure 2:**
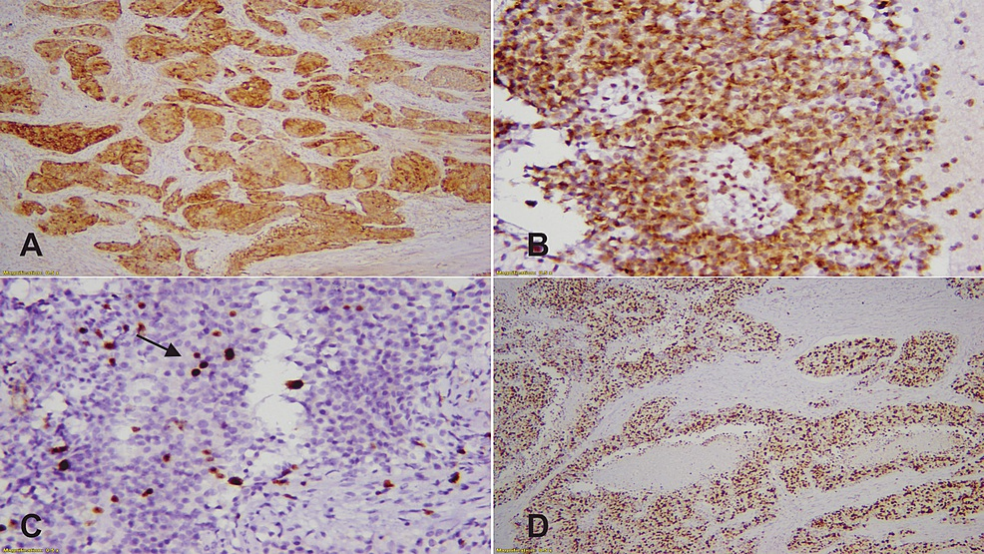
(A) The image shows diffuse cytoplasmic staining of Synaptophysin. (B) Diffuse granular cytoplasmic staining of Chromogranin A. (C) Ki67 shows > 3% proliferation index, focal nuclear staining (black arrow). (D) The Image shows > 50% Ki67 proliferation index in NEC. NEC: neuroendocrine carcinoma

To prepare standard hematoxylin and eosin (H&E) glass slides, tissue fixed in 10% buffered formalin was processed in an automated tissue processor (Peloris, Leica, Germany) and then cut at 3 µm thickness (Figures 1A-1D). Subsequently, the immunohistochemical stains were applied on selected paraffin-embedded blocks by using the automated technique with the following primary monoclonal antibodies: Ki-67 (mouse anti-human antibody, clone MIB-1), Synaptophysin (mouse anti-human antibody, clone DAK-SYNAP), and Chromogranin A (mouse anti-human antibody, DAK-A3), all from Dako, Glostrup, Denmark (Figures 2A-2D).

The cases were reviewed by two pathologists with expertise in gastroenteropancreatic pathology. The tumor grade was evaluated by the Ki-67 proliferation index and mitotic count (in the hot spot areas) according to the WHO classification (2019). In cases where Ki67 proliferation index and mitotic activity were discordant, higher numerical value was considered to assign grade. The AJCC TNM classification system was used for staging (8th edition).

The data were analyzed using SPSS version 22 (IBM Corp., Armonk, NY, USA). In descriptive analyses, numerical variables were reported as means and standard deviations, whereas frequencies or categorical variables were expressed as percentages. Chi square test was used for analysis of GEP-NET grade with patient’s demographic data and clinicopathological features including pT stage and site.

## Results

The current study included 87 GEP-NET cases, consisting of 40 biopsies and 47 resection specimens. There were 49 males (56.3%) and 38 females (43.7%) ranging in age from 11 to 80 years (mean: 45.7± 16.4). The majority of them were < 50 years (49 cases, 56.3%).

**Figure 3:**
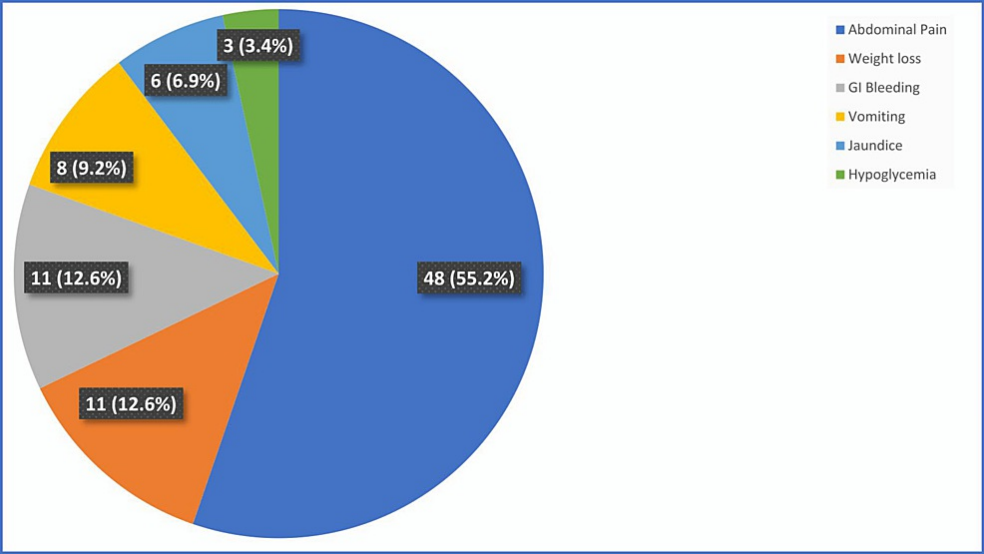
Frequency of clinical symptoms in GEP-NETs patients (n=87) GEP-NETs, gastroenteropancreatic neuroendocrine tumors

The majority of GEP-NETs (85 cases, 96.5%) were nonfunctional while functional tumors (insulinoma) were found in three (3.4%) of the patients. The most common presentation was abdominal pain (48 cases, 55.2%), weight loss, and GI bleeding (11 cases, 12.6% each) (Figure 3).

**Figure 4:**
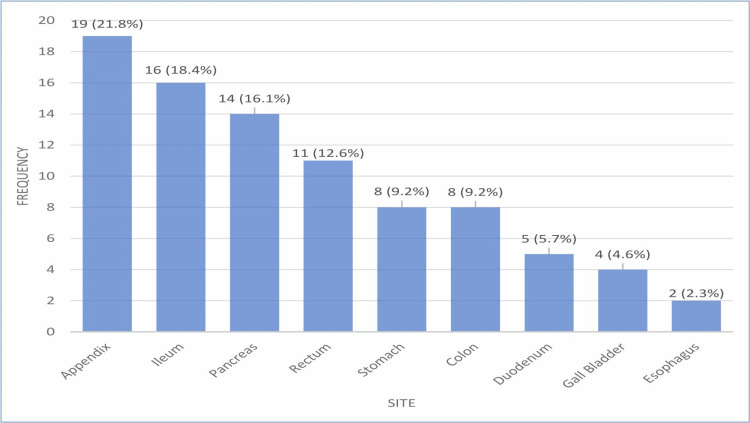
Distribution of GEP-NETs at various anatomical sites (n=87) GEP-NETs, gastroenteropancreatic neuroendocrine tumors

Appendix (19 cases, 21.8%) and ileum (16 cases, 18.4%) were the most common tumor sites, followed by the pancreas (14 cases, 16.1%) and rectum (11 cases, 12.6%). Other sites include the stomach and colon (eight cases, 9.2% each), the duodenum (five cases, 5.7%), the gallbladder (four cases, 4.6%), and the esophagus (two cases, 2.3%) (Figure 4).

The most frequent grade was NET grade 1 (54 cases, 62%), followed by NET grade 2 (21 cases, 24.1%), NET grade 3 (nine cases, 10.3%), which included both NET (three cases) and NEC (six cases), and MINENs (three cases, 3.5%). Immunohistochemical stain Synaptophysin was positive in 73 out of 87 cases (83.9%) while Chromogranin A was positive in 34 out of 87 cases (39.1%).

In the current study, 47 resection specimens out of a total of 87 specimens (excluding biopsies) could be pathologically staged (pT). Eight (17.2%) of the patients were staged as pT1, 12 (25.5%) were staged as pT2, 17 (36.1%) patients were staged as pT3 and 10 (21.2%) were staged as pT4. Only 12 (25.5%) out of 47 resection specimens were presented with nodal metastasis.

A comparative analysis of the GEP-NETs grade with gender, age, and the pT stage are summarized in Table 1. In our study, it was found that tumor grade did not substantially correlate with gender, age, or pT stage, indicating that these were independent pathological parameters. Among the clinicopathological parameters, tumor site and grading were found to have a strong correlation (p-value < 0.05) (Table 2). With the exception of one case of the appendix and four cases of ileum that was reported as NET grade 2, the majority of the tumors found in the appendix (18 out of 19) and ileum (11 out of 16) were NET grade 1 tumor. Only one ileum case was reported as NET grade 3. All four of the gallbladder cases, including three MINENS, were classified as grade 3.

**Table 1:** Comparative analysis of clinicopathological parameters and NET grade (n=87) *Chi-square test was applied; p-values = not statistically significant NET, neuroendocrine tumor; MINEN, mixed neuroendocrine-non-neuroendocrine neoplasm

Clinicopathological Parameters	NET Grade 1	NET Grade 2	NET Grade 3	MINEN	Total n (%)	P-value*
Age group (years)	(n=54)	(n=21)	(n=9)	(n=3)	Total (n=87)	0.219
<50	32 (59.2%)	13 (61.9%)	3 (33.3%)	1 (33.3%)	49 (56.3%)	
>50	22 (40.7%)	8 (38%)	6 (66.6%)	2 (66.6%)	38 (43.7%)	
Gender	(n=54)	(n=21)	(n=9)	(n=3)	Total (n=87)	0.712
Male	29 (53.7%)	12 (57.1)	5 (55.5%)	3 (100%)	49 (56.3%)	
Female	25 (46.2%)	9 (42.8%)	4 (44.4%)	0 (0.00%)	38 (43.7%)	
Pathological Tumor (pT) stage	(n=32)	(n=8)	(n=5)	(n=2)	Total (n=47)	0.328
pT1	6 (18.7%)	2 (25%)	0 (0.00%)	0 (0.00%)	8 (17.2%)	
pT2	8 (25%)	2 (25%)	2 (40%)	0 (0.00%)	12 (25.5%)	
pT3	12 (37.5%)	2 (25%)	1 (20%)	2 (100%)	17 (36.1%)	
pT4	6 (18.7%)	2 (25%)	2 (40%)	0 (0.00%)	10 (21.2%)	

**Table 2:** Comparative analysis of tumor site and NET grade (n=87) *Chi-square test was applied; **p-value significant as < 0.05 NET: neuroendocrine tumor; MINEN: mixed neuroendocrine-non-neuroendocrine neoplasm

Tumor Site	NET Grade 1 (n=54)	NET Grade 2 (n=21)	NET Grade3 (n=9)	MINEN (n=3)	Total n=87	P-value*
Appendix	18(33.3%)	1 (4.76%)	0 (0.00%)	0 (0.00%)	19 (21.8%)	0.0001**
Ileum	11 (20.3%)	4 (19.0%)	1 (11.1%)	0 (0.00%)	16 (18.4%)	
Rectum	7 (12.9%)	3 (14.2%)	1 (11.1%)	0 (0.00%)	11 (12.6%)	
Pancreas	6 (11.1%)	7 (33.3%)	1 (11.1%)	0 (0.00%)	14 (16.1)	
Stomach	3 (5.55%)	2 (9.52%)	3 (33.3%)	0 (0.00%)	8 (9.2%)	
Colon	4 (7.40%)	2 (9.52%)	2 (22.2%)	0 (0.00%)	8 (9.2%)	
Duodenum	4 (7.40%)	1 (4.76%)	0 (0.00%)	0 (0.00%)	5 (5.7%)	
Gall Bladder	0 (0.00%)	0 (0.00%)	1 (11.1%)	3 (100%)	4 (4.6%)	
Esophagus	1 (1.85%)	1 (4.76%)	0 (0.00%)	0 (0.00%)	2 (2.3%)	

## Discussion

GEP-NETs are an uncommon heterogeneous category of tumors that develop from the secretory cells of the gastrointestinal tract and pancreatic endocrine systems [7]. GEP-NETs can manifest as hormonally functional or nonfunctional tumors, with varying clinical characteristics depending on the site of origin [8].

According to the latest World Health Organization (WHO) 2019 classification, neuroendocrine neoplasms of the gastroenteropancreatic tract are categorized into well-differentiated neuroendocrine tumor (NET) G1, G2 and G3, poorly-differentiated neuroendocrine carcinoma (NEC) and mixed neuroendocrine-non-neuroendocrine neoplasms (MINENs) (Figure 3). Tumor proliferative activity and morphological parameters are used to grade the tumors. According to the Ki-67 index, the grades are G1, G2, and G3 as < 3%, 3%-20%, and > 20%, respectively. Likewise, tumors having mitotic rates of < 2 per 10 high-power fields (HPF) or 2mm² are categorized as G1, 2-20/HPF as G2, and > 20/HPF as G3 [9].

Genetic research over the past few years has revealed that extra-pancreatic neuroendocrine tumor cells, particularly those of the gastro-intestinal tract, have genetic alterations strikingly similar to those of the pancreas. Similar to pancreatic and pulmonary NECs, gastrointestinal NECs commonly have TP53 and RB1 mutations, although they are not present in NETs. Well-differentiated NETs are characterized by mutations in MEN1, DAXX, and ATRX [10].

According to the various studies on this topic conducted in other countries, a male predominance was observed, and our population also exhibited a similar trend. [2,5,6]. The majority of the patients were under the age of 50 (56.3%) in the present series. This finding, however, contradicted other studies, the most well known of which was conducted by Tughba et al., and showed opposite results [5].

Talking about the presenting symptoms, published literature revealed that the majority of patients had nonfunctional tumors, with abdominal pain being the most common symptom, rather than functional tumors causing symptoms such as hypoglycemic attack. [5,8]. A similar trend was observed in our population.

On reviewing the published data, the most common sites of GEP-NETs in the previous literature were the appendix and ileum [3,11,12], but the most recent data showed a shift toward the stomach being the most common site in the Turkish population [5,8]. However, in our population, the most common site was the appendix (21.8%), followed by the ileum (18.4%), and the stomach ranked 5th (9.2%). It is obvious that different nations may have different distribution patterns for GEP-NETs. This disparity can be most logically explained by differences in ethnicity, race and geographical location of the center [13].

According to a study by Tolga et al. on the Turkish population, NET grade 1 tumors made up the majority of GEP-NETs cases (61.8%), followed by NET grade 2 tumors (18.8%) and NET grade 3 tumors (19.4%), which included both NET grade 3 and NEC cases [14]. These findings were nearly similar to ours.

In a recent study by Yu-Chen et al., MINENs were reported in the stomach and intestines, followed by the pancreas [15]. In our population, only three (3.5%) cases of MINENs were found, and they were all gallbladder related (Figure 2D). Two of the three MINENS cases were found to have a high pathological tumor (pT3) stage. It was the most significant finding observed in our analysis, and it had not previously been reported in any study, with the exception of a few case reports. According to a few case reports, MINEN had been found in the gallbladder and this fact was consistent with our study [16,17]. Despite the fact that MINENs are more common than was previously postulated and that the morbidity and mortality rates of gastrointestinal MINENs are alarmingly rising, there is a scarcity of research and data [15].

GEP-NETs frequently manifest at advanced stages, as evidenced by 36.1% of patients having a high pathological tumor stage (pT3) and 25.5% with nodal metastases in the current study. This finding was in line with that of the study conducted in India by Megha et al. [6]. It justifies the fact that over 95% of patients had non-functional GEP-NETs that manifested relatively late, with symptoms of mass effect and a high pT stage.

The grading is one of the most important pathological parameters. A study conducted on the Turkish population by Tughba et al. revealed a comparison of the GEP-NETs grade with gender, age, pT stage, and tumor site. It was demonstrated that while tumor site was substantially correlated with grade, demographics and pT stage were independent pathological factors [5]. These findings were consistent with our study.

Due to the fact that these tumors are neuroendocrine in nature, they would stain positive for neuroendocrine markers such as Synaptophysin, Chromogranin A, CD56, and the novel marker INSM-1. Our research has some limitations, including the lack of patient follow-up data that prevents survival analyses and the study of disease recurrence. The results might not accurately represent the true prevalence of GEP-NET in other regions since the study was conducted at a single center.

## Conclusions

In our study, the appendix and ileum were the most common sites of GEP-NETs. Among the clinicopathological features, the primary site and tumor grade were shown to be substantially correlated, but there was no significant relationship between the tumor grade and the gender, age, or pT stage assessed. Our research will be helpful in providing epidemiological information regarding the clinicopathological characteristics of GEP-NETs in our population. We anticipate that our comprehensive analysis of GEP-NETs will improve doctors’ understanding of the tumors and lead to earlier detection and treatment for patients.
